# Physician Health Care Visits for Mental Health and Substance Use During the COVID-19 Pandemic in Ontario, Canada

**DOI:** 10.1001/jamanetworkopen.2021.43160

**Published:** 2022-01-21

**Authors:** Daniel T. Myran, Nathan Cantor, Emily Rhodes, Michael Pugliese, Jennifer Hensel, Monica Taljaard, Robert Talarico, Amit X. Garg, Eric McArthur, Cheng-Wei Liu, Nivethika Jeyakumar, Christopher Simon, Taylor McFadden, Caroline Gerin-Lajoie, Manish M. Sood, Peter Tanuseputro

**Affiliations:** 1 Clinical Epidemiology Program, Ottawa Hospital Research Institute, Ottawa, Ontario, Canada; 2 Department of Family Medicine, University of Ottawa, Ottawa, Ontario, Canada; 3 ICES, Ontario, Canada; 4 Department of Psychiatry, University of Manitoba, Winnipeg, Manitoba, Canada; 5 School of Epidemiology and Public Health, University of Ottawa, Ottawa, Ontario, Canada; 6 Department of Medicine, Western University, London, Ontario, Canada; 7 Department of Epidemiology and Biostatistics, Western University, London, Ontario, Canada; 8 Canadian Medical Association, Ottawa, Ontario, Canada; 9 Department of Medicine, University of Ottawa, Ottawa, Ontario, Canada; 10 Division of Nephrology, Department of Medicine, The Ottawa Hospital, Ottawa, Ontario, Canada

## Abstract

**Importance:**

Physicians self-report high levels of symptoms of anxiety and depression, and surveys suggest these symptoms have been exacerbated by the COVID-19 pandemic. However, it is not known whether pandemic-related stressors have led to increases in health care visits related to mental health or substance use among physicians.

**Objective:**

To evaluate the association between the COVID-19 pandemic and changes in outpatient health care visits by physicians related to mental health and substance use and explore differences across physician subgroups of interest.

**Design, Setting, and Participants:**

A population-based cohort study was conducted using health administrative data collected from the universal health system (Ontario Health Insurance Plan) of Ontario, Canada, from March 1, 2017, to March 10, 2021. Participants included 34 055 physicians, residents, and fellows who registered with the College of Physicians and Surgeons of Ontario between 1990 and 2018 and were eligible for the Ontario Health Insurance Plan during the study period. Autoregressive integrated moving average models and generalized estimating equations were used in analyses.

**Exposures:**

The period during the COVID-19 pandemic (March 11, 2020, to March 10, 2021) compared with the period before the pandemic.

**Main Outcomes and Measures:**

The primary outcome was in-person, telemedicine, and virtual care outpatient visits to a psychiatrist or family medicine and general practice clinicians related to mental health and substance use.

**Results:**

In the 34 055 practicing physicians (mean [SD] age, 41.7 [10.0] years, 17 918 [52.6%] male), the annual crude number of visits per 1000 physicians increased by 27%, from 816.8 before the COVID-19 pandemic to 1037.5 during the pandemic (adjusted incident rate ratio per physician, 1.13; 95% CI, 1.07-1.19). The absolute proportion of physicians with 1 or more mental health and substance use visits within a year increased from 12.3% before to 13.4% during the pandemic (adjusted odds ratio, 1.08; 95% CI, 1.03-1.14). The relative increase was significantly greater in physicians without a prior mental health and substance use history (adjusted incident rate ratio, 1.72; 95% CI, 1.60-1.85) than in physicians with a prior mental health and substance use history.

**Conclusions and Relevance:**

In this study, the COVID-19 pandemic was associated with a substantial increase in mental health and substance use visits among physicians. Physician mental health may have worsened during the pandemic, highlighting a potential greater requirement for access to mental health services and system level change.

## Key Points

**Question:**

Has the incidence of physicians seeking outpatient care for mental health and substance use changed during the COVID-19 pandemic?

**Findings:**

In a cohort study of 34 055 physicians, the rate of outpatient visits for mental health and substance use increased on average by 13% per physician during the first 12 months of the pandemic compared with the prior 12 months.

**Meaning:**

These findings suggest that the COVID-19 pandemic is associated with greater mental health services use among physicians.

## Teaser

This cohort study examines the levels of mental health and substance use visits by physicians during the COVID-19 pandemic.

## Introduction

Studies have documented high levels of mental health and substance use concerns among physicians.^1,2,3,4^ The emergence of the COVID-19 pandemic poses additional risks to the mental health of physicians.^5^ In addition to the general societal disruption from the COVID-19 pandemic, physicians face specific occupational stressors, including a potentially greater risk of exposure to SARS-CoV-2, with consequent concerns over personal health and infecting family, friends, and colleagues^6^; inadequate personal protective equipment^7^; rapid practice changes including loss of income; and high, and at times overwhelming, workloads.^8^ There are additional concerns about trauma arising from moral distress when physicians face difficult decisions regarding the allocation of scarce resources or balancing their needs and those of their patients.^9^

During the pandemic, health care workers, including physicians, have self-reported high levels of stress, anxiety, and depression. Surveys of physicians in China (n = 493) and New York (n = 282) during the first months of the COVID-19 pandemic found that 42.8% of respondents had at least mild symptoms of depression^10^ and 41% screened positive for depression.^11^ A survey of approximately 1300 Canadian physicians during late 2020 found that 62% reported being quite or extremely stressed most days.^12^ Two cross-sectional surveys (n = 1407 and n = 2649) of Ontario physicians found that the proportion of respondents reporting being completely burned-out (a strong correlate of mental health issues^13^) increased from 10.6% in March 2020 to 14.0% in March 2021.^14^ However, to date, studies on physicians’ mental health during the pandemic have used small cross-sectional samples that,^10,11,12,14^ combined with low response rates (eg, <10% in the Ontario surveys), raise concerns of whether they represent all physicians. In addition, few studies have directly compared changes in mental health before and during the COVID-19 pandemic, limiting our understanding of whether surveys are capturing a pandemic-related change in physicians’ mental health or the prepandemic baseline.^1,2,3,4^ To our knowledge, no studies have examined changes in mental health care service use among physicians and by physician subgroups. To address these gaps, we used health administrative data to examine population-level changes in outpatient visits related to mental health and problematic substance use (a potential marker of elevated stress and maladaptive coping) among Canadian physicians before and during the COVID-19 pandemic.

## Methods

### Study Design and Setting

We conducted a cohort study of practicing physicians in Ontario, the most populous province in Canada (n = 14.7 million in 2020), using linked health administrative data from the Ontario Health Insurance Plan (OHIP), the province’s universal health care system. The data sets used in this study capture nearly all outpatient visits to physicians in Ontario. We identified physicians (n = 45 835) who registered between 1990 and 2018 with the College of Physicians and Surgeons of Ontario, a requirement to practice medicine in Ontario. Physicians were assessed for cohort eligibility between March 1, 2017, and March 10, 2021, and were excluded from follow-up during periods in which they were not living in Ontario, OHIP eligible, or died (n = 11 780 excluded from the entire study). Physicians were linked to health care visits using unique, encoded identifiers obtained from the College of Physicians and Surgeons of Ontario by a small, specialized group at ICES (formerly known as the Institute for Clinical and Evaluative Sciences). All identifying information was removed (deidentified) before data were sent to the study team. The eMethods 1 in the Supplement provides additional methodological details on data linkage and cohort creation. Our study time frame (March 11, 2020, to March 10, 2021) covers the first wave of COVID-19 and state of emergency in Ontario (March-May 2020), along with a phased reopening during a period of declining and then relatively low case incidence of COVID-19 (June-September 2020). This period was followed by a second wave starting in mid-September 2020, which continued until March 2021.^15^ This project was conducted under section 45 of Ontario’s Personal Health Information Protection Act, which allows ICES to collect personal health information without consent for the purpose of health system evaluation and improvement, and approved by ICES’s Privacy and Legal Office. This study followed the Strengthening the Reporting of Observational Studies in Epidemiology (STROBE) reporting guideline observational studies.

### Outcomes

Our primary outcome was an outpatient visit (including virtual care and telemedicine) by a physician to another physician related to mental health or substance use. We defined mental health and substance use–related outpatient visits using a previously validated definition.^16^All visits to psychiatrists were considered mental health and substance use–related visits. Visits to a primary care physician were considered related to mental health and substance use if a suitable mental health and substance use diagnostic or fee code was included (eTable 1 in the Supplement provides further details). As a secondary outcome, we captured all outpatient (eg, for any cause) visits by physicians (including virtual care and telemedicine visits) to another primary care or specialist physician to account for possible overall changes in the health-seeking behavior by physicians during the pandemic.

### Physician Subgroups and Covariates

Demographic characteristics (age and sex), urban or rural residence,^17^ and previous mental health history (≥1 mental health and substance use outpatient, emergency department [ED], or hospitalization encounters in the prior 2 years) were included as covariates and examined as subgroups. Data on individual race and ethnicity are not available within ICES. Physician specialty information was obtained from the College of Physicians and Surgeons of Ontario registration and the ICES Physician Database (eTable 2 in the Supplement provides a full breakdown of specialties). We identified physicians who provided direct acute care in the ED or inpatient setting for patients with suspected or confirmed COVID-19 using physician billing location and diagnostic codes (DX COVID). Residents and fellows do not submit billing claims and were excluded from this analysis. We sorted physicians into 3 categories (0, 1-5, and ≥6 patients cared for in the first year of the COVID-19 pandemic). The latter 2 categories were selected to create 2 equally sized groups of physicians who provided a lower and higher quantity of care for patients with COVID-19.

### Statistical Analysis

We used a seasonally adjusted autoregressive integrated moving average model applied to biweekly (14-day) rates of outpatient mental health and substance use visits (79 intervals before the pandemic) to forecast the expected number of outpatient mental health and substance use visits during the pandemic (26 intervals) with 95% CIs. The denominator for the biweekly rate was the number of physicians alive and eligible for OHIP coverage during the 2-week period. We selected the best-fitting model, specified as (0,0,0) × (0,1,1)_26_, based on the lowest values for the Akaike information criterion, and present the results graphically. We ran a separate autoregressive integrated moving average model in which the denominator was the number of outpatient all-cause visits to a primary care physician or specialist during the 2-week period. The model was specified as (1,0,1) × (1,1,0)_26_. The eMethods 2 in the Supplement provides details related to model selection for both autoregressive integrated moving average models.

We then analyzed changes in the number of mental health and substance use visits per physician by comparing the rates and proportions during the first year of the pandemic with the 12 months before the pandemic. Using the individual physician as the unit of analysis, we conducted generalized estimating equations with a Poisson distribution. The dependent variable was the number of mental health and substance use visits for each physician in each of the time periods. The independent variable of interest was included as a binary variable, interpreted as the rate of mental health and substance use visits during vs before the COVID-19 pandemic. Generalized estimating equations model the average number of mental health and substance use visits while accounting for the within-physician correlation between time periods. Physicians were compared with themselves if they were eligible in both time periods (96.3%). Physicians who were eligible in only one period were compared with the physician average in the other time period. We included an interaction term between the COVID-19 pandemic variable and each subgroup indicator to conduct exploratory analyses of differences by physician characteristics. To examine whether changes in visits were related to a change in the number of physicians with a visit, changes in frequency of repeat visits, or both, we report the proportion of physicians in each time period with 1 or more visits.

## Results

### Study Population

Our study included 34 055 physicians practicing in Ontario between 2017 and 2021; Of these, the mean (SD) age was 41.7 (10.0) years, 17 918 were men (52.6%), 16 137 were women (47.4%), and 32 841 (96.4%) lived in an urban region. A wide variety of physician specialties was represented, with the most common specialty being family medicine (10 561 [31.0%]). A total of 5839 physicians (17.1%) had 1 or more mental health visits in the 2 years before the start of the pandemic. During the first year of the pandemic, 2935 of 26 465 (11.1%) physicians (residents and fellows not examined) cared for at least 1 patient with suspected or confirmed COVID-19 in the ED or inpatient setting (Table 1).

**Table 1.** Characteristics of Physicians in 2019 and 2020^a^

Year (No. of physicians)	No. (%)	
	2019 (n = 32 706)	2020 (n = 31 472)
Age, mean (SD), y	42.2 (10.1)	43.1 (10.1)
Age, y		
<50	24 795 (75.8)	22 903 (72.8)
≥50	7911 (24.2)	8569 (27.2)
Sex		
Female	15 596 (47.7)	15 069 (47.9)
Male	17 110 (52.3)	16 403 (52.1)
Rural home address		
Yes	1204 (3.7)	1208 (3.8)
No	31 204 (95.4)	30 014 (95.4)
Missing	298 (0.9)	250 (0.8)
Physician specialty		
Family medicine	10 347 (31.6)	10 140 (32.2)
General internal medicine and specialties	4090 (12.5)	3953 (12.6)
Psychiatry	1548 (4.7)	1513 (4.8)
Anesthesia	1176 (3.6)	1149 (3.7)
Critical care/emergency medicine	1578 (4.8)	1551 (4.9)
Surgery	3377 (10.3)	3214 (10.2)
Pediatrics	1334 (4.1)	1280 (4.1)
Other	2936 (9.0)	2793 (8.9)
Trainee/first years in practice	5305 (16.2)	5007 (15.9)
Missing	1015 (3.1)	872 (2.8)
No. of ED or hospitalized patients with suspected or confirmed COVID-19 treated by physician during first year of pandemic^b^		
0	NA	23 530 (88.9)
1-5	NA	1540 (5.8)
≥6	NA	1395 (5.3)
Mental health visit in the past 2 y		
Yes	5706 (17.4)	5758 (18.3)
No	27 000 (82.6)	25 714 (81.7)

Abbreviation: ED, emergency department.

^a^ Characteristics of physicians were obtained annually on March 11 or at the time of their first eligibility in the calendar year.

^b^ Residents and fellows could not be associated with the care of patients suspected or confirmed to have COVID-19 (care was assigned through billing claims) and were excluded from this analysis.

### Changes in Mental Health and Substance Use Visits

**Figure 1.**
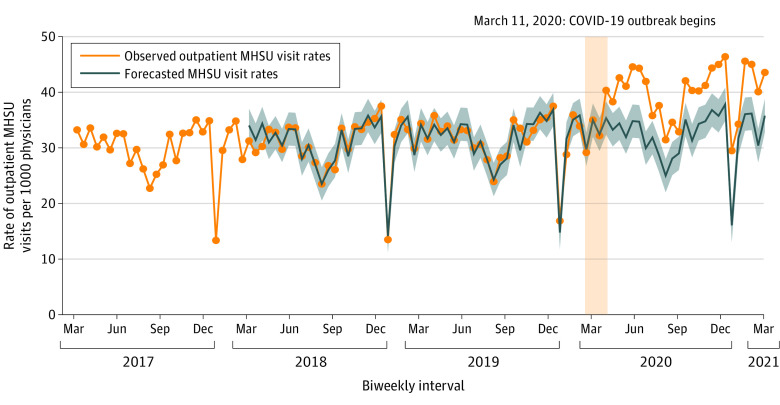
Biweekly (14-day) Number of Outpatient Mental Health and Substance Use (MHSU) Visits by Physicians per 1000 Physicians Between March 1, 2017, and March 9, 2021 The vertical yellow column represents the declaration of the COVID-19 pandemic on March 11, 2020. The forecasted numbers and 95% CIs were generated from an autoregressive integrated moving average model, which was specified as (0,0,0) x (0,1,1)_26_.

Figure 1 shows the biweekly number of outpatient mental health and substance use visits by a physician per 1000 physicians between March 1, 2017, and March 9, 2021. Before the COVID-19 pandemic, seasonal declines in visits occurred during December and between late July to early September. During the pandemic, mental health and substance use visits continued to follow the same seasonal patterns but were globally increased with 39.8 (95% CI, 37.8-41.8) biweekly visits per 1000 physicians during the pandemic compared with 30.8 (95% CI, 29.7-31.8) prepandemic.

**Figure 2.**
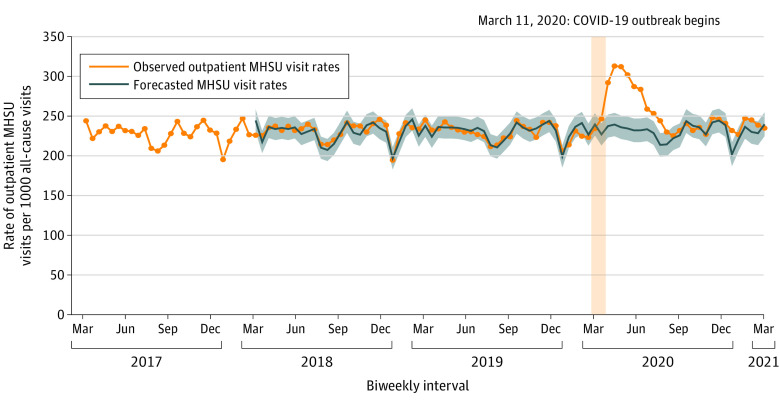
Biweekly (14-day) Number of Outpatient Mental Health and Substance Use (MHSU) Visits by Physicians per 1000 Outpatient All-Cause Physician Visits Between March 1, 2017, and March 9, 2021 The vertical yellow column represents the declaration of the COVID-19 pandemic on March 11, 2020. The forecasted numbers and 95% CIs were generated from an autoregressive integrated moving average model, which was specified as (1,0,1) x (1,1,0)_26_.

Figure 2 shows the biweekly number of outpatient mental health and substance use visits by a physician per 1000 all-cause outpatient visits between March 1, 2017, and March 9, 2021. On average, 23.0% (95% CI, 22.7-23.2) of all-cause outpatient visits by physicians were due to mental health and substance use before the COVID-19 pandemic. During the first 5 months of the pandemic, 28.3% (95% CI, 26.4-30.2) of the visits were related to mental health and substance use. In the following 7 months of the pandemic, the proportion of all-cause outpatient visits due to mental health and substance use returned to prepandemic levels owing to an increase in all-cause outpatient visits by physicians.

Table 2 presents the diagnostic codes associated with mental health and substance use visits by physicians. In both time periods, over 65% of mental health and substance use visits were due to anxiety and approximately 15% were related to a mood disorder. Visits related to anxiety and adjustment reactions had the largest increases during the COVID-19 pandemic; increases in visits related to mood disorders or other mental health conditions were smaller. Visits related to social and economic problems and alcohol and drug use declined or were unchanged.

**Table 2.** Counts of Outpatient Mental Health and Substance Use-Related Codes by Physicians During the First 12 Months of the COVID-19 Pandemic Compared With 12 Months Earlier^a^

Code type	Count of codes (% of all codes)^b^		% Change in codes
	Pre-COVID-19	During COVID-19	
Total codes	26 266	31 936	22.6
Mental health codes	23 574 (89.8)	29 460 (92.3)	25.6
Anxiety, somatoform, dysthymia, dissociative, psychosomatic	17 470 (66.5)	22 072 (69.1)	26.3
Adjustment reaction	919 (3.5)	1308 (4.1)	42.3
Mood disorders	4104 (15.6)	4868 (15.2)	18.6
Other mental health codes	1081 (4.1)	1212 (3.8)	12.1
Economic and social problem codes	741 (2.8)	627 (2.0)	−15.4
Drug and alcohol use codes	1951 (7.4)	1849 (5.8)	−5.2

^a^ Pre-COVID-19 pandemic: March 11, 2019, to March 10, 2020; during COVID-19 pandemic: March 11, 2020, to March 10, 2021.

^b^ Codes include visits to psychiatrists and family/general practice. There are more outpatient mental health and substance use visits than codes as all visits to psychiatrist (regardless of code) were included.

Table 3 compares the crude number (per 1000 physicians) of mental health and substance use visits in the first 12 months the COVID-19 pandemic with the prior 12 months and presents adjusted incident rate ratios (aIRRs) for the increase in visits per physician during the COVID-19 period. Before the pandemic, rates of mental health and substance use visits were higher in women compared with men and urban compared with rural physicians. We observed substantial variation in rates of mental health and substance use visits among specialty types. Psychiatrists had the highest rate of annual visits (3441.5 per 1000 physicians), and surgeons had the lowest rates of visits (370.9 visits per 1000 physicians). In addition, most visits (86.3%) before the pandemic were by physicians with a history of a mental health visit in the preceding 2 years.

**Table 3.** Outpatient Mental Health and Substance Use-Related Visits by Physicians During the First 12 Months of the COVID-19 Pandemic Compared With 12 Months Earlier^a^

Variable	Pre-COVID-19 pandemic		During COVID-19 pandemic		Crude % change in rates of visits	Incident rate ratio (95% CI)^b^
	No. of outpatient mental health and substance use visits (%)	No. of visits per 1000 physicians	No. of outpatient mental health and substance use visits (%)	No. of visits per 1000 physicians		
Overall	26 666	816.8	32 627	1037.46	27.0	1.13 (1.07-1.19)
Sex						
Female	16 489 (61.8)	1059.3	20 463 (62.7)	1358.8	28.3	1.16 (1.09-1.25)
Male	10 177 (38.2)	595.8	12 164 (37.3)	742.2	24.6	1.07 (0.97-1.18)
Age, y						
<50	20 816 (78.1)	841.2	24 633 (75.5)	1076.3	28.0	1.14 (1.07-1.21)
≥50	5850 (21.9)	740.5	7994 (24.5)	933.7	26.1	1.10 (0.97-1.24)
Rural^c^						
Yes	543 (2.0)	451.5	767 (2.4)	635.5	40.7	1.30 (0.84-2.00)
No	26 023 (97.6)	835.4	31 721 (97.2)	1057.6	26.6	1.13 (1.06-1.19)
Specialty						
Family medicine	7110 (26.7)	687.6	4362 (27.9)	890.3	29.5	NC^d^
General internal medicine and specialties	2284 (3.2)	558.7	1393 (8.9)	733.4	31.3	NC^d^
Psychiatry	5324 (20.0)	3441.5	6429 (19.7)	4252.0	23.5	NC^d^
Anesthesia	501 (1.9)	427.1	854 (2.6)	743.3	74.0	NC^d^
Critical care/emergency medicine	1097 (4.1)	696.1	1299 (4.0)	838.1	20.4	NC^d^
Surgery	1250 (4.7)	370.9	1480 (4.5)	460.6	24.2	NC^d^
Pediatrics	806 (3.0)	604.2	1024 (3.1)	801.3	32.6	NC^d^
Other	1631 (6.1)	556.1	1861 (5.7)	666.6	19.9	NC^d^
Trainee/first years in practice	5638 (21.1)	1069.4	6617 (20.3)	1323.4	23.7	NC^d^
Missing	1025 (3.8)	1011.9	1144 (3.5)	1313.4	29.8	NC^d^
No. of ED or hospitalized patients with suspected or confirmed COVID-19 treated by physician^e^						
0	19 366 (92.5)	823.6	26 010 (92.4)	1022.2	24.1	1.13 (1.06-1.21)
1-5	756 (3.6)	491.2	996 (3.8)	647.2	31.7	1.23 (0.89-1.70)
≥6	802 (3.8)	574.9	978 (3.8)	701.1	21.9	1.05 (0.77-1.43)
Mental health visit in the past 2 y						
Yes	23 014 (86.3)	4041.1	27 731 (85.0)	4821.1	19.3	0.99 (0.92-1.07)^f^
No	3652 (13.7)	135.5	4896 (15.0)	190.5	40.6	1.72 (1.60-1.85)

Abbreviations: ED, emergency department; NC, not completed.

^a^ Pre-COVID-19 pandemic: March 11, 2019, to March 10, 2020; during COVID-19 pandemic: March 11, 2020, to March 10, 2021.

^b^ Incident rate ratio comparing count of visits (per physician) during the first 12 months of COVID-19 to the previous 12 months. Analyses were adjusted for sex, age, rurality, specialty, and prior mental health.

^c^ Rows do not sum to 100% owing to missing status on location.

^d^ Incident rate ratios not completed for individual specialties.

^e^ Residents and fellows could not be associated with the care of patients suspected or confirmed to have COVID-19 (care was assigned through billing claims) and were excluded from this analysis.

^f^ Interaction test for subgroup difference was significant (*P* < .001) only for those with and without a mental health visit in the past 2 years.

During the COVID-19 pandemic, the crude annual number of visits per 1000 physicians increased by 27.0% (816.8 prepandemic to 1037.5 during the pandemic). The rate of visits per physician increased significantly during the pandemic (aIRR, 1.13; 95% CI, 1.07-1.19). Increases were observed across multiple subgroups with no significant differences between men (aIRR, 1.07; 95% CI, 0.97-1.18) and women (aIRR, 1.16; 95% CI, 1.09-1.25) (*P* = .10), older (aIRR, 1.10; 95% CI, 0.97-1.24) and younger physicians (aIRR, 1.14; 95% CI, 1.07-1.21) (*P* = .55), and urban (aIRR, 1.13; 95% CI, 1.06-1.19) and rural physicians (aIRR, 1.30; 95% CI, 0.84-2.00) (*P* = .72), and for physicians who provided infrequent (aIRR, 1.23; 0.89-1.70), more frequent (aIRR, 1.05 (0.77-1.43), or no (aIRR, 1.13; 95% CI, 1.06-1.21) (*P* = .72) care for COVID-19 patients in the ED or hospital during the first year of the pandemic. The relative increase in the rate of visits by physicians without a mental health and substance use history was significantly greater (aIRR, 1.72; 95% CI, 1.60-1.85) than by physicians with a mental health and substance use history (aIRR, 0.98; 95% CI, 0.92-1.07). The absolute proportion of physicians requiring 1 or more mental health and substance use visits increased by 1.1% from 12.3% (n = 4027) in the 12 months before the pandemic to 13.4% (n = 4225) in the first 12 months of the pandemic (odds ratio for 1 or more visits per physician during vs before the pandemic of 1.08; 95% CI, 1.03-1.14).

## Discussion

In a population-level cohort study of 34 055 unique physicians, the annual rate of outpatient mental health and substance use visits by physicians increased by 27.0% (from 816.8 to 1037.5 per 1000 physicians) during the first 12 months of the COVID-19 pandemic compared with the preceding 12 months. After adjusting for demographic and physician characteristics and a history of health care use related to mental health, visits increased on average by 13% per physician (aIRR, 1.13; 95% CI, 1.07-1.19). Although not statistically significant, greater increases in the frequency of visits were observed among female physicians and physicians in rural areas. We did not observe a large difference in changes in visits between physicians who did or did not care for patients with suspected or confirmed COVID-19 in the ED or hospital. The largest relative increases in the rate of visits occurred in physicians without a mental health and substance use history, which was significantly greater than increases in physicians with a mental health and substance use history.

We observed increases in mental health and substance use visits during the COVID-19 pandemic that, consistent with surveys finding high levels of self-reported anxiety, depression, and stress in physicians during the pandemic,^10,11,12^ may indicate worsening physician mental health. Although OHIP diagnostic codes for mental health visits are not specific, we observed the largest increases in visits for anxiety and adjustment reactions, which would be expected to begin and/or worsen in the context of highly stressful circumstances, such as a pandemic. Our findings suggest that these increases are related to both an increase in the total number of physicians who accessed mental health services and an increase in the number of physicians with multiple mental health visits. These increases were particularly evident among physicians without a pre-existing mental health diagnosis. Together, these findings suggest that generally, physicians have displayed resiliency during the pandemic, but a small group of physicians may have developed very high new mental health care needs during the pandemic, which are possibly related to pandemic-specific stressors.

Our findings may also be explained by reduced barriers to access for health care and mental health services among physicians during the COVID-19 pandemic. In response to the pandemic there was a large expansion of virtual care options in Ontario.^18^ It is possible that physicians with both physical and mental health concerns that predated the pandemic increased their health services use owing to this change (eg, appointments are easier to schedule and less visible and thus less stigmatized).^19^ Although we found that the proportion of total outpatient visits owing to mental health increased during the pandemic, we also observed that outpatient visits not related to mental health by physicians increased during the latter half of the first year of the pandemic. Whether these increases in overall visits are capturing acute, pandemic-related declines in health, increased opportunities for physicians to access care for prepandemic conditions, or a combination is unclear. Further work exploring changes in patterns of care in physicians during the COVID-19 pandemic, including virtual visits, is needed.

Prior work examining health care workers’ psychological well-being during viral outbreaks, including 8 studies during the COVID-19 pandemic that were included in a meta-analysis, has reported that prolonged contact with infected patients was a risk factor for negative psychological outcomes.^20^ In contrast, our results found that increases in mental health and substance use visits during the pandemic did not differ substantially between individuals who either did or did not provide acute care for patients with COVID-19. There are a number of possibilities for this discrepancy. First, physicians providing acute care for patients with COVID-19 had lower rates of mental health and substance use visits before the pandemic. This finding suggests that this group of physicians may have increased resilience to mental health conditions, a greater reluctance to seek mental health care, or a combination that continued during the pandemic. Second, owing to high workloads, this group of physicians may not have had time to seek mental health care during the pandemic.

Although not the primary purpose of our study, we noted patterns of mental health visits by physicians in the pre-COVID-19 period. First, consistent with findings in the general population,^21^ female physicians had a higher rate of mental health and substance use visits compared with male physicians. Second, among all specialties, psychiatrists were the most likely to have mental health and substance use visits, and surgeons and anesthesiologists were the least likely to have visits. Differences in visits by specialty likely reflect both the burden of mental health problems and differences in patterns of care seeking. For example, high numbers of visits by psychiatrists might be partly explained by studies showing that many psychiatrists report routinely attending therapy to improve their personal and professional growth.^22^ In contrast, it has been suggested that surgeons have reduced care-seeking behaviors owing to a greater perception of stigma surrounding mental health.^23^ Prior research has found that physicians experience similar barriers to seeking health care as the general population, including time constraints.^24^ It is possible that physicians with more demanding schedules are less able to access outpatient services and may therefore benefit from alternative modes of access (ie, telehealth or virtual care).

Although the COVID-19 pandemic may have exacerbated physician mental health concerns, findings from this study and previous work have documented that many of these concerns predate the start of the pandemic.^1,2,3,4^ Consequently, interventions to improve the mental health of physicians should focus on both acute stressors related to COVID-19 and prepandemic factors. A recent report by the Ontario Medical Association, based on physician self-identified priorities, recommends that system reforms, such as reducing documentation and administrative work, ensuring fair and equitable pay for all work, and improving work life balance, are critical to protecting the mental health and well-being of physicians.^14^ Our work highlights additional solutions, which may include improving access to mental health services for physicians (ie, continuing the delivery of virtual mental health care).

### Strengths and Limitations

Strengths of this study include a large sample size (>30 000 physicians), a longitudinal design, and an analytic approach that controls for prepandemic changes in outpatient mental health and substance use visits. In addition, the use of administrative health data from a universal health care system captures virtually all health care visits, which increases the generalizability of this study to other regions. This study also has limitations. First, physicians have low levels of care-seeking behaviors related to mental health and substance use,^25,26^ and encounters related to mental health and substance use by physicians may be incorrectly coded as due to other reasons by providers owing to concerns over discrimination from regulatory bodies and stigma. Consequently, our study has likely only captured more severe outcomes and may miss other patterns of changes in important outcomes, such as physician burnout. We expect that this bias and source of misclassification would be consistent over time. Second, although this study used a validated and highly sensitive definition to identify mental health and substance use visits, the available diagnostic algorithm is not fully reliable at identifying the exact cause of a visit (eg, depression vs anxiety) and changes in rates of specific conditions should be interpreted with caution.^16^ Third, during the COVID-19 pandemic, several organizations have increased access to mental health services for physicians delivered by nonphysician professionals.^27^ Our study did not include such services, which may lead to underestimates of increases in physician mental health care during the pandemic. Fourth, physicians faced a variety of occupation-specific stressors (eg, balancing childcare and patient obligations, substantial practice disruptions) beyond providing acute care for individuals with COVID-19. Further research should investigate the relationship between such stressors and physician mental health.

## Conclusions

This study noted that, during the first 12 months of the COVID-19 pandemic, physicians in Ontario experienced an increase in outpatient visits related to mental health and substance use. These findings may signal that the mental health of physicians has been negatively affected by the pandemic. Future research should focus on longer term outcomes associated with the pandemic and explore associated risk and protective factors for physicians’ mental health to better target interventions.

## Notes

Supplement. **eMethods 1.** Cohort and Data Sources Information **eTable 1.** Definition of MHSU Visits and List of OHIP Codes **eTable 2.** List of All Physician Specialties **eMethods 2.** ARIMA Model Building Summary
