# Supplementary material for: Physician Health Care Visits for Mental Health and Substance Use During the COVID-19 Pandemic in Ontario, Canada
Source: JAMA Netw Open. 2022 Jan 21;5(1):e2143160. doi: 10.1001/jamanetworkopen.2021.43160 (PMC8783265; doi:10.1001/jamanetworkopen.2021.43160)
Supplement: Supplement. — eMethods 1. Cohort and Data Sources Information eTable 1. Definition of MHSU Visits and List of OHIP Codes eTable 2. List of All Physician Specialties eMethods 2. ARIMA Model Building Summary [file jamanetwopen-e2143160-s001.pdf]

Supplementary Online Content

Myran DT, Cantor N, Rhodes E, et al. Physician health care visits for mental health and substance use during the COVID-19 pandemic in Ontario, Canada. *JAMA Netw Open*. 2022;5(1):e2143160. doi:10.1001/jamanetworkopen.2021.43160

eMethods 1. Cohort and Data Sources Information

eTable 1. Definition of MHSU Visits and List of OHIP Codes

eTable 2. List of All Physician Specialties

eMethods 2. ARIMA Model Building Summary

This supplementary material has been provided by the authors to give readers additional information about their work.

© 2022 Myran DT et al. *JAMA Network Open*.

eMethods 1. Cohort and Data Sources Information

Physician Linkage between CPSO and ICES

Physicians were linked to health care visits using unique, encoded identifiers from the CPSO. Deterministic followed by probabilistic linkage (based on name, date of birth, and sex) was performed by a small, specialized group at ICES (formerly known as the Institute for Clinical and Evaluative Sciences). All identifying information was removed before data were sent to the study team. This is done to mitigate any privacy breaches. ICES is an independent, non-profit research institute that houses routinely collected health data from Ontario's publicly funded health care system. ICES is a prescribed entity under section 45 of Ontario's Personal Health Information Protection Act. Section 45 authorizes ICES to collect personal health information, without consent, for the purpose of health system evaluation and improvement. Projects conducted under section 45, by definition, do not require review by a specific institutional research ethics board. This project was conducted under section 45, and approved by ICES's Privacy and Legal Office.

Data Sources

Physician demographic and speciality information was collected through the CPSO Registration Database and from the ICES Physicians Database. Data on physician characteristics and health care use were obtained through the following linked databases at ICES: 1) the Ontario Registered Persons Database, which captured demographic information including age and sex; 2) the postal code conversion file which contains information on the rurality of an individual's home address; 3) the OHIP Claims Database, which captured all outpatient claims for mental health visits, including virtual visits, in Ontario; and 4) the National Ambulatory Care Reporting System (NACRS), Ontario Mental Health Reporting System (OMHRS), and Discharge Abstract Database (DAD), which were used to capture acute care use as part of the definition for past history of mental health or substance use.

© 2022 Myran DT et al. *JAMA Network Open*.

eTable 1. Definition of MHSU Visits and List of OHIP Codes
List of all mental health and substance use (MHSU) related OHIP billing and fee codes used to determine outpatient visits. We identified MHSU visits using the methods from the Mental Health and Addictions Scorecard and Evaluation Framework indicator (MHASEF). We telemedicine and virtual care visits when a physicians' OHIP claim included either 1) a mental health-related diagnostic or fee code in combination with a telemedicine flag or 2) a mental health-related diagnostic and a corresponding virtual fee code.

**Mental Health and Substance Use Codes**	**Description of Code**
Diagnostic Codes	
295	Schizophrenia
296	Manic-depressive psychoses, involutional melancholia
297	Other paranoid states
298	Other psychoses
300	Anxiety neurosis, hysteria, neurasthenia, obsessive-compulsive neurosis, reactive depression
301	Personality disorders
302	Sexual deviations
306	Psychosomatic illness
307	Habit spasms, tics, stuttering, tension headaches, anorexia nervosa, sleep disorders, enuresis
309	Adjustment reaction
311	Depressive disorder
897	Economic problems
898	Marital difficulties
899	Parent-child problems
900	Problems with aged parents or in-laws
901	Family disruption / divorce
902	Education problems
904	Social maladjustment
905	Occupational problems
906	Legal problems
909	Other problems of social adjustment
291	Alcoholic psychosis, delirium tremens, Korsakov's psychosis
292	Drug psychosis
303	Alcoholism
304	Drug dependence
Fee Codes	
A680	Initial assessment – substance abuse
K680	Substance abuse – extended assessment

© 2022 Myran DT et al. *JAMA Network Open*.

To avoid double-counting, patients with multiple mental health-related claims from one provider on the same day only contributed to one visit. However, we allowed patients who saw multiple providers in one day to contribute one visit per provider (i.e., a patient who saw a primary care physician for a mental health-related reason and a psychiatrist on the same day would contribute two visits). We excluded tobacco use (OHIP diagnostic code 305) and mental health codes related to pediatric presentations (OHIP Diagnostic Codes 313, 314, 315, and 319) from the MHASEF indicator.

© 2022 Myran DT et al. *JAMA Network Open*.

eTable 2. List of All Physician Specialties
List of all physician specialties available in the CPSO dataset and the categories assigned for the purpose of this study. The trainee/recent graduate group was defined as CPSO registrants with missing specialty information, where the year of graduation from medical school was between 2013 and 2018.

**Study Specialty Groups**	**CPSO Specialties**
Family Medicine	GP/FP, CCFP – FAMILY MEDICINE, CPSO – FAMILY MEDICINE
General Internal Medicine and Medicine Specialties*	INTERNAL MEDICINE, RCPSC – INTERNAL MEDICINE, NEUROLOGY, CARDIOLOGY, RCPSC – NEUROLOGY, RESPIROLOGY, GASTROENTEROLOGY, HEMATOLOGY, MEDICAL ONCOLOGY, ENDOCRINOLOGY, NEPHROLOGY, , RHEUMATOLOGY, GERIATRICS MEDICINE, INFECTIOUS DISEASES, RCPSC – RESPIROLOGY, RCPSC – MEDICAL ONCOLOGY, RCPSC – MEDICAL ONCOLOGY, RCPSC – CARDIOLOGY, RCPSC – NEPHROLOGY, RCPSC – ENDOCRINOLOGY AND METABOLISM, RCPSC – GASTROENTEROLOGY, RCPSC – HEMATOLOGY, RCPSC – INFECTIOUS DISEASES, CPSO – INTERNAL MEDICINE, CPSO – CARDIOLOGY, CPSO – MEDICAL ONCOLOGY, RCPSC – GERIATRIC MEDICINE, RCPSC – RHEUMATOLOGY, CPSO – HEMATOLOGY, CPSO – RESPIROLOGY, CPSO – GASTROENTEROLOGY, CPSO – INFECTIOUS DISEASES, CPSO – NEUROLOGY, CPSO – ENDOCRINOLOGY AND METABOLISM, RCPSC – CLINICAL PHARMACOLOGY AND TOXICOLOGY, CLINICAL PHARMACOLOGY
Surgery	GENERAL SURGERY, OBSTETRICS AND GYNECOLOGY, ORTHOPEDIC SURGERY, RCPSC – GENERAL SURGERY, OPHTHALMOLOGY, RCPSC – ORTHOPEDIC SURGERY, OTOLARYNGOLOGY, RCPSC – OBSTETRICS AND GYNECOLOGY, UROLOGY, PLASTIC SURGERY, RCPSC – OPHTHALMOLOGY, RCPSC – UROLOGY, NEUROSURGERY, CARDIAC SURGERY, RCPSC – NEUROSURGERY, RCPSC – OTOLARYNGOLOGY – HEAD AND NECK SURGERY, RCPSC – PLASTIC SURGERY, RCPSC – CARDIAC SURGERY, THORACIC SURGERY, VASCULAR SURGERY, RCPSC – VASCULAR SURGERY, PEDIATRIC SURGERY, CPSO – OBSTETRICS AND GYNECOLOGY, RCPSC – PEDIATRIC GENERAL SURGERY, RCPSC – THORACIC SURGERY, CPSO – CARDIAC SURGERY, CPSO – GENERAL SURGERY, CPSO – ORTHOPEDIC SURGERY, CPSO – OTOLARYNGOLOGY – HEAD AND NECK SURGERY, RCPSC – CARDIOVASCULAR AND THORACIC SURGERY, RCPSC

© 2022 Myran DT et al. *JAMA Network Open*.

	– GENERAL SURGICAL ONCOLOGY, CPSO – OPHTHALMOLOGY, OTHER PGT – SURGICAL ONCOLOGY, RCPSC – PAEDIATRIC SURGERY, COLORECTAL SURGERY, SURGICAL ONCOLOGY GYNECOLOGIC ONCOLOGY, RCPSC – GYNECOLOGIC ONCOLOGY
Psychiatry	PSYCHIATRY, RCPSC – PSYCHIATRY, CPSO – PSYCHIATRY, RCPSC – CHILD AND ADOLESCENT PSYCHIATRY, RCPSC – FORENSIC PSYCHIATRY, RCPSC – FORENSIC PSYCHIATRY, RCPSC – GERIATRIC PSYCHIATRY, CPSO – FORENSIC PSYCHIATRY, CHILD & ADOLESCENT PSYCHIATRY, FORENSIC PSYCHIATRY, GERIATRIC PSYCHIATRY
Anaesthesia	ANESTHESIOLOGY, RCPSC – ANESTHESIOLOGY, CPSO – ANESTHESIOLOGY
Critical Care/Emergency Medicine	CRITICAL CARE, RCPSC – CRITICAL CARE MEDICINE, PEDIATRIC CRITICAL CARE, NO CERT – CRITICAL CARE MEDICINE, EMERGENCY MEDICINE, RCPSC – EMERGENCY MEDICINE, F.P./EMERGENCY MEDICINE, CCFP – FAMILY MEDICINE (EMERGENCY MEDICINE), PEDIATRIC EMERGENCY MEDICINE, RCPSC – PAEDIATRIC EMERGENCY MEDICINE, CPSO – EMERGENCY MEDICINE
Pediatrics	PEDIATRICS, RCPSC – PEDIATRICS, CPSO – PEDIATRICS, PEDIATRIC CLINICAL IMMUNOLOGY, PEDIATRICS CARDIOLOGY, RCPSC – PAEDIATRICS HAEMATOLOGY/ONCOLOGY, PEDIATRIC GASTROENTEROLOGY, PEDIATRIC NEUROLOGY, PEDIATRIC NEUROLOGY, PEDIATRIC HEMATOLOGY, PEDIATRIC INFECTIOUS DISEASES, PEDIATRIC NEPHROLOGY, PEDIATRIC RESPIROLOGY, ADOLESCENT MEDICINE, DEVELOPMENTAL PEDIATRICS, PEDIATRIC ENDOCRINOLOGY, PEDIATRIC RHEUMATOLOGY
Other	CPSO – PHYSICAL MEDICINE AND REHABILITATION, RCPSC – PHYSICAL MEDICINE AND REHABILITATION, PALLIATIVE MEDICINE, PHYSICAL MEDICINE AND REHAB,MEDICAL MICROBIOLOGY, RCPSC – PAIN MEDICINE, PAIN MEDICINE, RCPSC – MEDICAL GENETICS, RCPSC – OCCUPATIONAL MEDICINE, OCCUPATIONAL MEDICINE, MATERNAL FETAL MEDICINE, NO CERT – MATERNAL-FETAL MEDICINE, CPSO – NEONATAL-PERINATAL MEDICINE, NEONATAL/PERINATAL MEDICINE, RCPSC – MATERNAL FETAL MEDICINE, REPRODUCTIVE ENDOCRINOLOGY, RCPSC – GYNECOLOGIC

© 2022 Myran DT et al. *JAMA Network Open*.

	REPRODUCTIVE ENDOCRINOLOGY & INFERTILITY, CPSO – DERMATOLOGY, CLINICAL IMMUNOLOGY, RCPSC – CLINICAL IMMUNOLOGY AND ALLERGY, RCPSC – MEDICAL MICROBIOLOGY, MEDICAL GENETICS, RCPSC – DERMATOLOGY, DERMATOLOGY, DIAGNOSTIC RADIOLOGY, RCPSC – DIAGNOSTIC RADIOLOGY, CPSO – DIAGNOSTIC RADIOLOGY, NO CERT – NEURORADIOLOGY, RCPSC – NEURORADIOLOGY, RCPSC – PAEDIATRIC RADIOLOGY, CPSO – PAEDIATRIC RADIOLOGY, FELLOW, FRCPC – FRCPC, ANATOMICAL PATHOLOGY, RADIATION ONCOLOGY, RCPSC – ANATOMICAL PATHOLOGY, RCPSC – RADIATION ONCOLOGY, FRCSC – FRCSC, GENERAL PATHOLOGY, LAB MEDICINE, NUCLEAR MEDICINE, HEMATOLOGICAL PATHOLOGY, RCPSC – HEMATOLOGICAL PATHOLOGY, RCPSC – GENERAL PATHOLOGY, RCPSC – NUCLEAR MEDICINE, RCPSC – NEUROPATHOLOGY, MEDICAL BIOCHEMISTRY, NEUROPATHOLOGY, CPSO – ANATOMICAL PATHOLOGY, RCPSC – FORENSIC PATHOLOGY, CPSO – RADIATION ONCOLOGY, CPSO – FORENSIC PATHOLOGY, CPSO – LABORATORY MEDICINE, FRCPSC – FRCPC(MSC), CPSO – GENERAL PATHOLOGY, CPSO – NEUROPATHOLOGY, RCPSC – MEDICAL BIOCHEMISTRY, FORENSIC PATHOLOGY, PEDIATRIC RADIOLOGY, COMMUNITY MED./PUBLIC HEALTH, RCPSC – COMMUNITY MEDICINE, RCPSC – PUBLIC HEALTH AND PREVENTIVE MEDICINE, CPSO – PUBLIC HEALTH AND PREVENTIVE MEDICINE

*Note: In Canada, General Internal Medicine (GIM)) is considered a subspeciality of internal medicine, and requires an extra year of training following three core years of internal medicine. General internists do not provide direct primary care and are only accessible by referral from another physician. For more information see: https://link.springer.com/article/10.1007/s11606-016-3891-z

© 2022 Myran DT et al. *JAMA Network Open*.

eMethods 2. ARIMA Model Building Summary

General explanation:

We used the ARIMA procedure in SAS to produce descriptive output to identify an ARIMA model to forecast expected rates. We only used data up to the point of forecasting to build the model. We used time series output to determine the need for an ARIMA model and the differencing required to stabilize the series. We used autocorrelation function (ACF) and partial autocorrelation function (PACF) plots, and white noise autocorrelation tests to guide the selection of model terms. Terms were retained if they were significant. We identified better performing models using AIC. Model building stopped when there was no indication that further terms needed to be added (e.g. no patterns in the ACF/PACF and no significant autocorrelations).

Model terms:

Non-seasonal terms: (p,q,d)
Seasonal terms: $(P,Q,D)_s$
Seasonal factor: S = 26 (26 biweekly periods in a year).

Model 1:

We fit an ARIMA model to the biweekly rates of physician mental health visits (per 1,000 physicians) and compared the predicted rates post COVID to observed values.

Model building steps:

- Step 1: identify if the series is stationary.
  - Time series plot show a seasonal trend – suggests series should be seasonally differenced.
- Step 2: make the series stationary.
  - Seasonally differencing the series (D=26) eliminated seasonal trend.
  - Sinusoidal pattern in ACF – suggests an AR term could improve the model.
  - Sinusoidal pattern in PACF – suggests an MA could improve the model.
- Step 3: non-seasonal AR and MA terms.
  - Non-seasonal autoregressive term (p=1) and non-seasonal moving average term (q=1) are non-significant when added to the model and did not improve model AIC.
  - White noise probabilities at higher order lags were approaching significance - suggests a seasonal AR or MA term could improve the model.
- Step 4: seasonal AR and MA terms.
  - Model with seasonal MA term (Q = 26) improved model AIC greater than model with seasonal AR term. AIC did not improve with both terms in the model.
  - ACF and PACF plots, and white noise tests do not suggest additional model terms.
- Step 5: model 1 specification
  - $(0,0,0)x(0,1,1)_{26}$

Model 2

- Step 1: identify if the series is stationary.
  - Time series plot show a seasonal trend – suggests series should be seasonally differenced.
- Step 2: make the series stationary.

© 2022 Myran DT et al. *JAMA Network Open*.

- Seasonally differencing the series (D=26) eliminated the seasonal trend.
    - Sinusoidal pattern in ACF – suggests an AR term could improve the model.
    - Sinusoidal pattern in PACF – suggests an MA could improve the model.
- Step 3: non-seasonal AR and MA terms.
    - Model with both AR (p=1) and MA (q=1) terms improved the model AIC.
    - White noise probabilities at higher order lags are significant - suggests a seasonal AR or MA term could improve the model.
- Step 4: seasonal AR and MA terms.
    - Model with seasonal AR term improved the AIC.
    - ACF and PACF plots, and white noise tests do not suggest additional model
- Step 5: model specification
    - $(1,0,1)x(1,1,0)_{26}$

Methodological guides:

- Hyndman, Rob and Athanasopoulos, George. Foreacasting: Principles and Practice, 2nd edition. Last accessed August 17th, 2021. https://otexts.com/fpp2/
- Nau, Robert. Statistical forecasting: notes on regression and time series analysis. Last accessed August 17th, 2021. https://people.duke.edu/~rnau/411home.htm
- Penn State Eberley College of Science – Applied time series analysis. Last accessed August 17th, 2021. https://online.stat.psu.edu/stat510/lesson/4
- SAS/ETS 13.2 User's Guide – The ARIMA Procedure. Last accessed August 17th, 2021. https://support.sas.com/documentation/onlinedoc/ets/132/arima.pdf
- Shaffer et al. Interrupted time series analysis using autoregressive integrated moving average (ARIMA) models: a guide for evaluating large-scale health interventions. *BMC Medical Research Methodology*. 2021, 21:58. https://doi.org/10.1186/s12874-021-01235-8

© 2022 Myran DT et al. *JAMA Network Open*.
